# Resumption of Elective Orthopaedic Surgery in the US Epicenter of COVID-19: Overcoming the Continuous Challenges

**DOI:** 10.3389/fsurg.2022.842591

**Published:** 2022-04-19

**Authors:** Eugene S. Krauss, MaryAnne Cronin, Nancy Dengler, Debra Schulman, Marie Marzano, Ayal Segal

**Affiliations:** ^1^ Department of Orthopaedic Surgery, Syosset Hospital, Northwell Health, Syosset, NY, United States; ^2^ Zucker School of Medicine, Hofstra/Northwell, Hempstead, NY, United States; ^3^ New York Orthopaedic and Spine Center, Great Neck, NY, United States

**Keywords:** COVID-19, elective surgery, orthopedic surgery, total joint arthroplasty, pandemic, thromboprophylaxis

## Abstract

On 1 March 2020, New York State confirmed its first case of COVID-19. An explosive progression of hospitalizations ensued, and all elective surgeries were cancelled between 23 March and 13 May 2020 per federal and state mandate. Upon return to elective surgery in May, 2020, the hospital found itself navigating uncharted territory. The unpredictability of the post-pandemic environment has required the healthcare team to constantly reassess and revise processes to ensure optimal patient outcomes, as well as safe practices for staff providing perioperative care. Health care professionals must continue to remain adaptable and amenable to constant change.

## Introduction

Syosset Hospital is part of the Northwell Health system, New York’s largest health care provider. Northwell Health comprises 23 hospitals and more than 830 outpatient facilities (1). On 1 March 2020, New York State confirmed its first case of COVID-19, and Northwell Health saw its first patient on 8 March, 2020. An explosive progression of hospitalizations ensued, and all elective surgeries were cancelled between 23 March and 13 May 2020 per federal and state mandate (2, 3). Although all metropolitan area hospitals were still caring for patients with severe symptoms from COVID-19, the need to address urgent surgery was mounting. On 29 April 2020, New York State released detailed requirements for resumption of elective surgery (4). Northwell Health chose two sites to become “non-COVID” in order to recommence urgent elective procedures. As discussed by Krauss et al., the task of returning to elective orthopaedic surgery during a pandemic placed the hospital into uncharted territory (5). This manuscript describes the evolution of protocols once our center resumed surgery. The challenges of the past 2 years have required constant reassessment and revision of processes to ensure optimal patient outcomes, as well as safe practices for staff providing perioperative care.

### Preoperative Period

**Figure 1:**
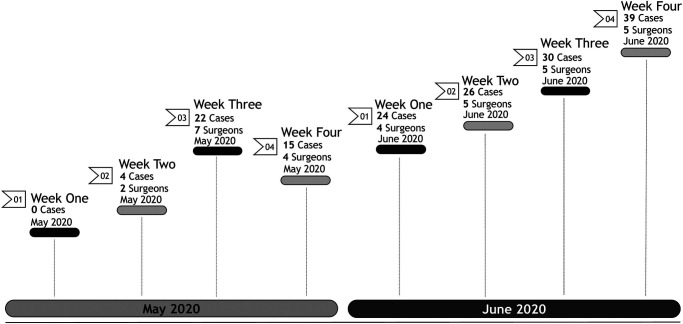
Procedure volume upon return to elective surgery. May–June 2020.

Upon return in May 2020, prioritization of scheduling patients for surgery was initially performed based on a patient acuity scale of 1 (elective, non-urgent), 2 (semi-urgent), 3 (urgent), to 4 (emergent). The higher the score, the more severe the patient’s pain and decreased ability to perform activities of daily living. The ongoing pandemic surge created a challenge for patients who required multiple presurgical clearances as many physician offices were still relying primarily on telemedicine. Therefore, these patients were not considered candidates for surgery during the initial reopening. Additionally, as the toll of the pandemic was especially prevalent in subacute rehabilitation centers, any patient not prepared for home discharge was also forced to delay surgery. Resumption of surgery began slowly upon return, especially that there were multiple “visiting surgeons” whose home base was at a different Northwell site, but urgently required operating room time to address unmet patient needs (Figure 1).

The pandemic resulted in the cancellation of all mandatory in-person preoperative arthroplasty education classes. Just prior to the pause in elective surgery, the department had developed an online education class as an alternative option to the in-person class. The class included a PowerPoint slide deck with a professional voiceover. After reopening, an additional slide deck was created to describe all new hospital COVID-19 protocols and restrictions for patients preparing for surgery. A postcard containing the website link to the education class located in the patient resources portal was distributed to all patients at the time their surgery was scheduled. Patients were then contacted by an Orthopaedic Clinical Program Manager to ensure they had watched the education video, to review the information provided, and to answer any questions.

### Presurgical Testing

Although the hospital was terminally cleaned and deemed “COVID-free” to facilitate a safe environment for elective surgeries, the forced suspension of on-site Pre-Surgical Testing (PST) created a different challenge. A comprehensive medical history was obtained by the PST nurse practitioner via telephone interview with the patient and/or family member. This practice was problematic as the nurses were either unable to get in contact with the patient or the patient did not have adequate time to complete the assessment over the telephone. To overcome these issues, the PST department began mailing scheduled appointments to the patients to assign a “phone visit” so the history could be completed as accurately as possible. Patients were provided the timeframe for the call along with the phone number the nurse would use for the call. The PST department’s contact information was also included if the patient needed to make any changes to their scheduled appointment. The physical portion of the presurgical evaluation was completed on the morning of surgery when the patient arrived to the preoperative unit. Once the first surge of the pandemic had passed, PST visits resumed on-site (May 2021), and was a welcomed relief to the difficulties posed by the telemedicine method.

A COVID-19 polymerase chain reaction (PCR) nasal swab is performed on every patient 48 h prior to the day of surgery at a designated drive-through site on the hospital grounds. Preoperative nasal swabs are given priority status by the Northwell Health laboratory and results are received within eight hours to allow adequate time for patient notification. Patients are told to quarantine at home after the PCR swab is performed, and until arriving at the hospital on the day of surgery. COVID-19 vaccine status does not change this requirement. Only patients with a confirmed negative COVID-19 PCR result can proceed with their procedure. As per Northwell Health policy, patients testing positive can be rescheduled for surgery after ten days if they are symptom-free and cleared by their primary care physician. COVID-19 PCR testing is not repeated if the positive result occurred within ninety days.

Prescriptions for preoperative laboratory assessments were initially given to patients during the surgical booking process and patients were encouraged to have their blood work drawn at a Northwell-affiliated laboratory. However, based on insurance coverage this was not always possible. Collecting and reviewing the presurgical results of the blood work was difficult as they were received from various laboratories, requiring additional phone calls to obtain results via fax. This was a burdensome issue and added extra steps to the pre-surgical department workload. Finally, missing or abnormal labs needed to be repeated the day of surgery, increasing surgical cancellations. The impact on patient satisfaction and resource utilization due to the increased cancellation rate was incalculable. A pre-surgical debrief the day before surgery now focuses on these topics to minimize delay of the start of surgery as well as postponement of the procedure all together.

## Venous Thrombosis Assessment

Thrombocytopenia has been identified in COVID-19 infection, accompanied by elevated fibrinogen and D-dimer levels (6). Variable levels of prothrombin time (PT), activated partial thromboplastin time (aPTT) and International Normalized Ratio (INR) have been reported (7). Moreover, there is evidence of a direct association between D-dimer levels and poor prognosis in COVID-19 disease (8). This hallmark hypercoagulability quickly became a major concern to account for in the arthroplasty patient. As providers we are trained to practice based on prevailing medical evidence. However, there was a paucity of data surrounding COVID-19 infection and elective surgery, especially upon return to surgery in the Spring of 2020. To adequately capture this information, all patients are interviewed by the Clinical Program Manager preoperatively to assess any history of COVID-19 infection and associated severity of symptoms.

The Caprini Risk Score (CRS) is a tool that assigns a weighted number value in assessing risk for postoperative venous thrombosis. In 2019 we published a validation study proving the utility of the CRS following arthroplasty (9). Our orthopaedic department has been using the CRS to determine thromboprophylaxis for all arthroplasty patients since then. Patients with a CRS of 9 or less are considered low risk for VTE and prophylaxed with Aspirin enteric coated (ASA EC) 81 mg q12h. Patients with a CRS of 10 or greater are considered high risk and receive Apixaban 2.5 mg q12h. All thromboprophylaxis begins on POD1. Utilizing the CRS has led to exceptional outcomes of both efficacy and safety to prevent postoperative VTE following arthroplasty at our institution, with an annual VTE rate of 0.2% in 2020 (10).

The pandemic highlighted the necessity to revise our CRS scoring to include the known hypercoagulability associated with COVID-19 infection. Using a validated risk assessment tool has allowed the department to account for the added risk factors associated with the recovered COVID-19 patient. However, it is unknown how long hypercoagulability persists after infection. Dr. Joseph Caprini recommends that patients with a history of COVID-19 within the previous six months receive an additional two points on the CRS, as hypercoagulability is suspected to continue for 6 months post COVID-19 infection (11, 12). This is the process we have adopted for all arthroplasty patients; if a patient had COVID-19 infection in the prior 6 months with any symptoms (even if vaccinated and/or boosted), they are assigned an additional 2 points to their CRS.

**Figure 2:**
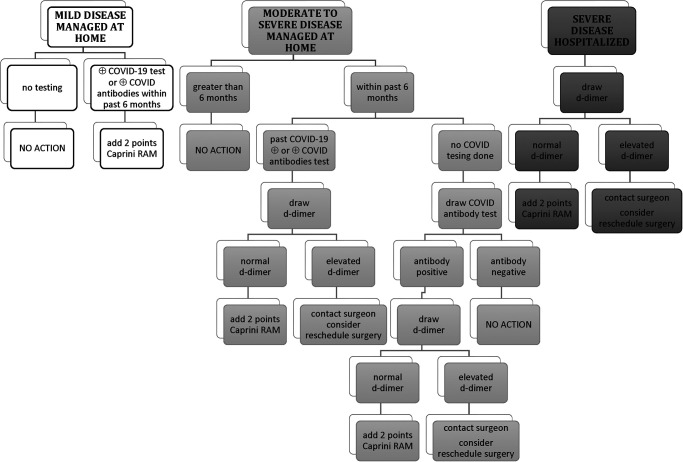
COVID-19 screening algorithm for elective orthopaedic surgery.

A questionnaire was developed in consultation with the orthopaedic surgeons and medical staff. Initially upon resumption of surgery, confirmation of COVID-19 illness was either by positive nasal swab or positive antibody testing. Since vaccination began, only positive nasal swabs are utilized for COVID testing. A COVID-19 Screening Algorithm was developed to guide our risk assessment of patients who tested positive (Figure 2). Given the known hypercoagulability associated with COVID-19 virus it was decided that patients who met criteria for severe disease would have a D-dimer drawn prior to surgery as part of their preoperative screening labs.

A total of 792 arthroplasty patients were evaluated for COVID-19 infection after the resumption of surgery in May through December 2020. (Table 1) Fourteen patients had confirmed COVID-19 disease within the six months prior to surgery. Five patients met the criteria for severe disease and a D-dimer was added to preoperative laboratory testing. None of the patients had an elevated D-dimer at the time of surgery. Of note, one patient had an elevated D-dimer (result = 395 ng/mL DDU, normal range ≤229 ng/mL DDU) 2 weeks prior to surgery. The D-dimer was repeated 7 days later in anticipation of the scheduled procedure and the result was within normal range (227 ng/mL DDU). This patient was approximately 6 months post COVID infection when this repeat D-dimer was performed. An additional “2 points” was added to the CRS for any patient reporting COVID-19 disease within the prior 6 months. These additional 2 points resulted in 7 patients moving from low risk to high risk for postoperative venous thromboembolism (VTE). The remaining 7 patients had no change in their treatment plan as their CRS remained less than 10.

**Table 1:** Arthroplasty procedures by month after resumption of elective surgery.

Month	Primary THA	Revision THA	Primary TKA	Revision TKA
May	13	1	25	2
Jun	62	0	55	2
Jul	44	3	66	5
Aug	43	2	68	3
Sep	36	4	68	3
Oct	35	3	86	1
Nov	30	2	51	2
Dec	23	1	53	0
**Total**	**286**	**16**	**472**	**18**

*The bold indicate total number of cases for each procedure May-December 2020*.

*THA, total hip arthroplasty; TKA, total knee arthroplasty*.

## Hospitalization

All postoperative arthroplasty patients were initially assigned a private room, although we have begun cohorting patients when necessary once the infection rate remained low in the community. All patients are required to wear a surgical mask when ambulating in the hallway or rehabilitation gym, and it is requested when hospital personnel are in their room, except during meals. The length of stay has been reduced to an average of 1.71 days. Although most patients are discharged on postoperative day (POD) 1 or 2, if the patient remains in the hospital, it is required to repeat the PCR every 4 days. Although quite rare, anyone found to test positive is immediately moved to a designated medical unit.

Traffic within the hospital has been dramatically reduced. No visitation was permitted when surgery initially resumed, until federal and state guidance eased restrictions on indoor gathering. Visitation remains fluid, responding to surges. Patients are strongly encouraged to watch a professionally taped video discussing educational information essential for safe recovery post arthroplasty. This video can be accessed via the patient television or iPad. The video has been made available on the internet for caregivers to view after patient discharge. During periods of suspended visitation, instructions are reviewed with the patient’s caregiver if requested via phone or Facetime in order to ensure a safe home discharge. Additionally, a nursing professional accompanies all patients to their car to address any questions or concerns posed by the caregiver. The surgeons’ offices took on the added responsibility of ordering durable medical equipment, as vendors have not been allowed in the hospital. Patient equipment (walker, commode) is now delivered to the patient’s homes prior to admission, or to the hospital lobby so they can be supplied to the patient prior to discharge.

With the expectation that all patients would be discharged home, especially early upon resumption of elective procedures, Home Care referrals greatly increased. Discharge to home increased from 64% to 98% over the past year in our arthroplasty patients. These additional referrals are performed by the care coordination department. A few patients voiced concerns about having a health care professional come into their home during the pandemic. Patients were assured that all medical personnel were required to double mask and wear appropriate personal protective equipment for home care visits. For the protection of staff, the home care department required their team to complete an additional COVID Screening Form, called “Discharge Planning COVID Screening Form”. This form risk assessed the patient’s home environment for potential COVID-19 exposures or infections among family members.

After a year of maintaining “non-COVID” hospital status for elective surgery, restrictions began to ease as the COVID-19 positivity rate dropped to 1.2% in Nassau County, New York on 1 May 2021 (13). On 3 May 2021 on-site PST appointments resumed. Visitation also returned but was limited to two visitors between the hours of 12 PM-6 PM with only one visitor at the bedside at a time. Upon reinstitution, all visitors were required to have their temperature checked when entering the hospital, and to wear surgical masks while visiting their loved one. As of 23 August 2021, upon Food and Drug Administration approval of the Pfizer COVID-19 vaccine, and amidst widespread circulation of the B.1.617.2 (Delta) variant, all visitors must either show proof of vaccination or proof of negative PCR/antigen COVID-19 test within 72 h of hospital visitation. Finally, with the rampant spread of the omicron variant in this part of the country, visitation was once again suspended as of 27 December 2021. On 1 March 2022, restricted visitation resumed with the same requirements as before.

Northwell Health treated more COVID patients than any other health system when the pandemic ravaged New York state in March 2020 (14). Chief Executive Officer Michael Dowling has been visible and vocal since the onset, and has reinforced the importance of preparedness and vigilance in battling this infection. When New York State announced a vaccine mandate for all healthcare workers, including a requirement to receive the first dose of a COVID-19 vaccine by 27 September 2021, senior leadership declared that Northwell would comply with this requisite (15). In advance of the 27 September deadline, with anticipated loss of more than 1,000 employees system wide, Northwell was prepared to deploy “staffing resources from a range of pipelines, including redeployed team members, per diems, agency staffing, new hires and students to where they were needed most” (16).

**Figure 3:**
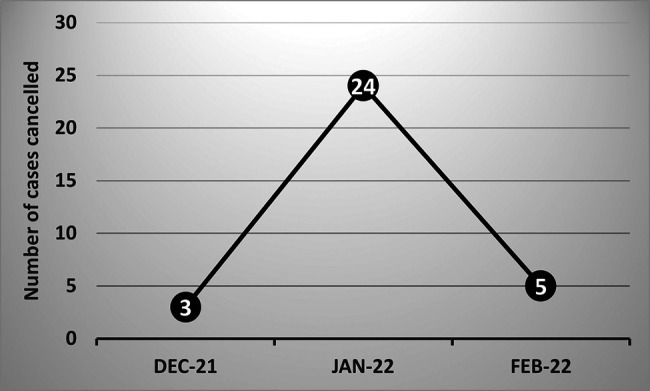
Cases cancelled following omicron surge.

Most recently, the December 2021–January 2022 omicron surge in New York added another hurdle for our arthroplasty program. With local COVID rates reported at 24%, although unofficially recognized to be about 50%, as many staff were testing positive as patients. Although approximately 50% of the hospital staff were unable to work, many elective cases were cancelled due to COVID-related patient issues, and the program continued uninterrupted through that time period (Figure 3).

Northwell Health is cognizant of the added burden that has been placed on its workforce, and the health system provides many avenues of assistance for employees especially challenged by the COVID-19 pandemic. Child care resources, crisis care reimbursement, emotional/well-being support, and physical health online series are some of the programs that have been made available to help maintain strong, viable personnel.

## Outcomes

Performing surgery in the unpredictable environment of a pandemic requires continuous re-evaluation of every policy and procedure. Federal and state guidance, and consequently work processes, are in constant flux. That being said, our goal remains the same, which is to provide excellent postoperative patient care following arthroplasty. Our annual outcomes have remained unchanged. In 2020 we maintained a postoperative VTE rate of 0.2%, which we attribute to revision of the CRS to incorporate the known hypercoagulability of COVID-19 infection. Additionally, surgical site infection rate remained low, at 0.2%.

## Conclusion

The response to the COVID-19 pandemic continues to evolve with the emergence of new viral variants. Health care professionals must continue to remain adaptable and amenable to constant change. Isolation of patients from family and friends during hospitalization combined with the innate need of healthcare professionals to provide excellent care has fostered a partnership with patients that did not exist pre-pandemic. We have become not only the patients’ caregivers, but their advocates as well. Despite the added daily workload to every team member, the ongoing revision of processes that continually change job responsibilities, and the challenges of work-life balance escalated by the pandemic, the multidisciplinary team has transformed into a finer ensemble than before. We are more kind, attentive, compassionate, and supportive towards patients, and towards each other. It is reflected in the comradery of our daily work, and in the excellent experience patients report after discharge. As a center of orthopaedic excellence, we have emerged from the unknown of the post-COVID-19 surge to a better place, continually striving to achieve optimal patient outcomes following arthroplasty.
